# Men with a history of commercial heterosexual contact play essential roles in the transmission of HIV-1 CRF55_01B from men who have sex with men to the general population in Guangxi, China

**DOI:** 10.3389/fcimb.2024.1391215

**Published:** 2024-08-23

**Authors:** Tong Luo, Fei Zhang, Huayue Liang, Dee Yu, Ping Cen, Shanmei Zhong, Cai Qin, Yuan Yang, Jiaxiao Jiang, Yanyan Liao, Mu Li, Rongjing Zhang, Zeyu Li, Zhifeng Lin, Li Ye, Hao Liang, Bingyu Liang

**Affiliations:** ^1^ Guangxi Key Laboratory of AIDS Prevention and Treatment, School of Public Health, Guangxi Medical University, Nanning, China; ^2^ Collaborative Innovation Centre of Regenerative Medicine and Medical Bioresource Development and Application Co-constructed by the Province and Ministry, Life Science Institute, Guangxi Medical University, Nanning, China; ^3^ Science and Technology Department, The First People’s Hospital of Qinzhou, Qinzhou, China; ^4^ International School of Public Health and One Health, Hainan Medical University, Haikou, China

**Keywords:** HIV-1, CRF55_01B, MSM, phylogenetic, Bayesian

## Abstract

**Background:**

There is increasing focus on HIV-1 CRF55_01B in China. However, there is limited information regarding the dissemination of CRF55_01B across different regions and populations in Guangxi. This study was performed to elucidate the evolutionary history of the introduction and dissemination of CRF55_01B in Guangxi.

**Methods:**

Molecular network and phylogenetic analyses were used to investigate the transmission characteristics of CRF55_01B in China. The analyses particularly focused on the cross-provincial spatial and temporal transmission patterns between Guangdong Province and Guangxi, as well as the transmission dynamics among different regions and populations within Guangxi.

**Results:**

In total, 2226 partial *pol* sequences of CRF55_01B strains sampled from 2007 to 2022 were collected, including 1895 (85.09%) sequences from Guangdong, 199 (8.94%) sequences from Guangxi, and 172 (7.59%) sequences from other provinces of China. Most people living with HIV in Guangxi were infected with HIV-1 through heterosexuals (52.76%). Among these, 19.10% had a history of commercial heterosexual contact (CHC) and 15.58% had a history of non-marital non-commercial heterosexual contact (NMNCHC). Overall, 1418 sequences were identified in the molecular network. Notably, the sequences from Guangdong Province were most closely linked to those from Guangxi. Phylogenetic analysis showed that CRF55_01B was first introduced from Shenzhen City to Nanning City around 2007. Subsequently, CRF55_01B established local transmission within Guangxi, with Nanning City serving as the transmission center from 2008 to 2017. After 2017, the CRF55_01B strain spread to other regions of Guangxi. Men who have sex with men (MSM) and men with a history of CHC have played a significant role in the transmission of CRF55_01B among different populations in Guangxi.

**Conclusions:**

This study provides evidence on the transmission trajectory of CRF55_01B among different regions and populations in Guangxi. Given the bridging role of men with a history of CHC in the dissemination of CRF55_01B from MSM to the general population, it is imperative to enhance surveillance among key populations to mitigate the secondary transmission of HIV-1.

## 1 Introduction

By the end of 2020, the number of people living with HIV (PLWH) in China was 1.22 million, with the vast majority infected with circulating recombinant forms (CRFs) (He, 2021). China is among the countries with a high prevalence of CRFs worldwide (Bbosa et al., 2019). According to the Los Alamos National Laboratory (LANL) HIV Database (https://www.hiv.lanl.gov/), more than 30 CRFs had been identified in China by the end of 2022. The high prevalence of CRFs will undoubtedly increase the complexity and challenges of HIV/AIDS prevention and control.

CRF55_01B is a novel HIV strain resulting from the recombination of CRF01_AE and B subtype variants. It was first identified among men who have sex with men (MSM) in Shenzhen City, China (Han et al., 2013). Although CRF55_01B has only been known for a decade and accounts for only 2.06% of the total number of HIV-1 infections in China (Wang et al., 2021), it has rapidly evolved and is considered the most important prevalent CRF by the Chinese Center for Disease Control and Prevention (Zhang et al., 2013), aside from CRF07_BC and CRF08_BC (Wei et al., 2021). Previous studies have indicated that the CRF55_01B strain originated in 2004 and was subsequently disseminated among MSM in 2013 (Zhao et al., 2014; Zai et al., 2020; Gan et al., 2021). Because of the rapidly developing railway system in China (Gan et al., 2021), the CRF55_01B strain has spread throughout the country through MSM, with a prevalence ranging from 1.5% to 12.5% in different regions (Wei et al., 2021). It is postulated that individuals infected with CRF55_01B may exhibit higher viral loads than those infected with CRF07_BC and CRF01_AE, and they may remain asymptomatic for an extended period because of a slower decline of their CD4^+^ T-cell count (Wei et al., 2021). Therefore, it is probable that CRF55_01B will exhibit more extensive transmission than CRF07_BC and CRF01_AE. CRF55_01B has reportedly spread from MSM to the heterosexual (HET) population and is progressively spreading within HET communities (Gan et al., 2021).

With the evolution of the HIV/AIDS epidemic, China has introduced two new categories of a non-marital heterosexual sexual contact (NMHC) history since 2014: commercial and non-commercial. In other words, a history of NMHC can now be further differentiated into a history of commercial heterosexual contact (CHC) or non-marital non-commercial heterosexual contact (NMNCHC). Previous research has suggested that HIV-1 transmission can occur among individuals with different infection routes. For instance, CRF55_01B can be transmitted from MSM to HETs, and CRF07_BC-N can be transmitted from MSM to injecting drug users and HETs (Gan et al., 2021; Gan et al., 2022). Few studies to date have been conducted to explore the relationships between HIV-1 transmission and diverse sexual contact histories.

Guangxi, located in the southwest region of China and sharing a border with Guangdong Province, was among the initial destination provinces to experience the transmission of CRF55_01B from Guangdong Province to other parts of China (Zai et al., 2020; Gan et al., 2021). Currently, there is no report on the transmission characteristics of CRF55_01B in different regions and populations with different sexual contact histories in Guangxi. In this study, data from the LANL HIV Database and the Guangxi Key Laboratory of AIDS Prevention and Control were used for molecular network and phylogenetic analyses to elucidate the transmission pathway of CRF55_01B in different regions and populations in Guangxi. The aim of this research is to provide valuable insights into local HIV/AIDS prevention and control efforts.

## 2 Materials and methods

### 2.1 Study population and sequences down load

From 1 January 2014 to 1 January 2023, we extracted viral nucleic acid from blood samples of HIV-infected individuals after their annual CD4^+^ T-cell count test. We then amplified the HIV *pol* partial region and sequenced the HIV *pol* sequence. Nearly 90% of the infected individuals in the sampled city undergo a CD4 ^+^ T-cell count test every year, allowing us to obtain these CRF55_01B *pol* sequences from 90% of the local HIV-infected individuals. Additional CRF55_01B *pol* sequences were obtained from the HIV database of the LANL. Each sequence represented an individual infected with HIV-1.

**Figure 1:**
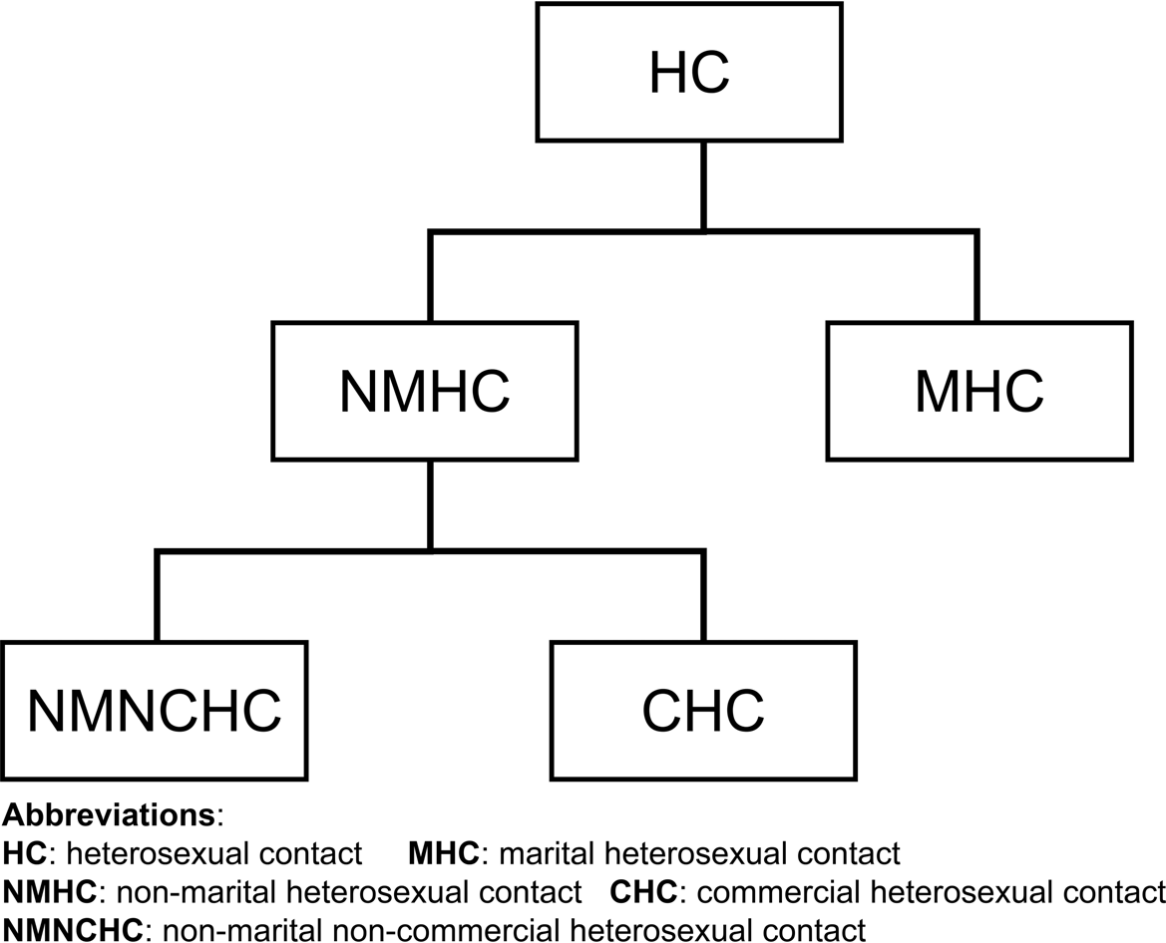
Five categories of heterosexual contact history.

Information on the sample year, sample city, sex, and transmission route were collected for sequences downloaded from the LANL HIV Database. For the collected sequences, data on the sample year, sample city, sex, marital status, and sexual contact history were obtained from the National Free Antiretroviral Treatment Program Database. HET sexual contact history includes five categories: HC, MHC, NMHC, CHC, and NMNCHC. These are depicted in **Figure 1**. Female sex workers were identified by staff from the local Center for Disease Control and Prevention.

All sequences were aligned to the HXB2 (2253-3312nt, 1060 base pairs) reference sequence using the online tool HIVAlign from the LANL HIV Database via the multiple alignment fast Fourier transform method. Recombination analysis was performed using the online tools jpHMM and RIP, which are also available from the LANL HIV Database. The maximum likelihood (ML) phylogenetic tree was constructed using FastML v3.1.1. Duplicate sequences, sequences with >5% mixed bases, and sequences with less than 1060 base pairs were excluded.

### 2.2 HIV-1 molecular network reconstruction

The genetic distance (GD) between two sequences was calculated using the Tamura-Nei 93 evolutionary model. The HIV-TRACE Transmission Cluster Engine (HIV-TRACE) was then employed to construct the molecular networks of CRF55_01B (Kosakovsky Pond et al., 2018), in accordance with the recommended GD threshold of 0.5% by the United States Centers for Disease Control and Prevention (National Center for HIV/AIDS VH, STD, and TB Prevention and Division of HIV/AIDS Prevention, 2018). The linkage degree is defined as the number of links between different regions in a molecular network.

### 2.3 Spatiotemporal transmission dynamics of CRF55_01B

Three subsets were selected from the entire sequence dataset (N = 2226) for Bayesian analysis. The first dataset was designed to determine the origin and introduction time of CRF55_01B into Guangxi. It consisted of 271 sequences from various provinces in China. The second dataset included sequences from both Guangxi and Guangdong Province and was designed to investigate the origin and spatiotemporal dynamics of CRF55_01B transmission, with geographical information specified down to the city level. The sequences in the first and second datasets were filtered based on representative samples from different years and regions within the branches of the ML tree constructed using FastTree. The third dataset (n = 138) exclusively contained sequences from Guangxi and was utilized to determine the transmission trajectories of CRF55_01B among individuals with different sexual contact histories. The sequences selected for this dataset were chosen based on their representation across different years and sexual contact histories identified in the ML tree. The drug resistance mutation sites were edited and excluded before the Bayesian analysis.

The ML trees were constructed using FastML v3.1.1 (Ashkenazy et al., 2012). Outlier sequences were then examined and excluded by TempEst v1.5.3 (Rambaut et al., 2016). To identify the origin and transmission time of CRF55_01B in Guangxi, a Bayesian asymmetric discrete phylogeographic model in BEAST v1.10.4 was employed (Suchard et al., 2018). A general time-reversible substitution model was used to enhance the migration rates and nucleotide substitution rates. The uncorrelated relaxed clock and the Bayesian skyline were employed as the tree prior. Markov chain Monte Carlo analysis was performed on the empirical tree for 200 million iterations, with sampling every 20,000 steps. This process was run twice independently in BEAST. Markov chain Monte Carlo analysis was also employed to estimate the most recent common ancestor (tMRCA). The outputs were analyzed using Tracer (Rambaut et al., 2018), and effective sampling size (ESS) values of >200 indicated sufficient convergence. LogCombiner was used to merge the log.txt and trees.txt files from the two runs to obtain the optimal maximum clade credibility (MCC) tree. After excluding the first 10% of runs as burn-in, the maximum clade credibility trees were summarized by TreeAnnotator v1.10.4 (Suchard et al., 2018) and then visualized in FigTree v1.7.2. The spatiotemporal dissemination of CRF55_01B was presented using a Bayesian stochastic search variable selection (BSSVS) procedure (Lemey et al., 2009). Bayes factors (BFs) (Lemey et al., 2009) were used to quantify statistical support, and the results were summarized with the widely-used tool SpreaD3 (Bielejec et al., 2016). In this study, BF < 3 was considered anecdotal evidence, 3 < BF ≤ 10 as substantial evidence, 10 < BF ≤ 30 as strong evidence, 30 < BF ≤ 100 as very strong evidence, and BF > 100 as decisive evidence.

## 3 Results

### 3.1 Socio-demographic characteristics

In total, 2226 CRF55_01B sequences were collected from 2007 to 2022. Of these, 2077 sequences were obtained from the LANL HIV Database, while the remaining 189 sequences were amplified in the present study. These sequences covered 19 provinces in China. Most of the sequences were obtained from Guangdong Province (1894, 85.09%), followed by Guangxi (199, 8.94%). Most sequences lacked information on the transmission route (56.38%, 1255/2226); 34.46% (767/2226) were from MSM. Among the sequences from Guangxi, most individuals had been infected with HIV-1 through HET (52.76%, 105/199), of whom 19.10% had a CHC history and 15.58% had an NMNCHC history (**Table 1**). More than half of the CRF55_01B-infected individuals in Guangxi came from Nanning City and Guigang City. A total of 90.45% of individuals infected with CRF55_01B were men, 69.85% had received more than 9 years of education, and 70.35% were divorced/widowed/unmarried. Further details are provided in **Supplementary Table S1** and **Supplementary Table S2**.

**Table 1:** Demographic information of individuals infected with CRF55_01B in Guangxi.

Variables	Total, n (%)
Sex	
Male	180 (90.45)
Female	19 (9.55)
Ethnicity	
Han	101 (50.75)
Other	98 (49.25)
Education, years	
≤9	33 (16.58)
>9	139 (69.85)
Unknown	27 (13.57)
Marital status	
Married/cohabitated	59 (29.65)
Divorced/widowed/unmarried	140 (70.35)
Sample year	
2009–2013	7 (3.52)
2014–2016	3 (1.51)
2017–2019	89 (44.72)
2020–2022	100 (50.25)
Sexual contact history	
MSM	94 (47.24)
HC	28 (14.07)
MHC	5 (2.51)
NMHC	3 (1.51)
CHC	38 (19.10)
NMNCHC	31 (15.58)
Sample location within Guangxi Province, region (cities)	
North (LZ)	12 (6.03)
East (HZ, WZ, YL)	8 (4.02)
Central (NN, GG)	114 (57.29)
South (QZ, BH)	28 (14.07)
West (BS, CZ, HC)	37 (18.59)
**Total**	199 (100)

MSM, men who have sex with men; HC, heterosexual contact; MHC, marital heterosexual contact; NMHC, non-marital heterosexual sexual contact; CHC, commercial heterosexual contact; NMNCHC, non-marital non-commercial heterosexual contact; LZ, Liuzhou; HZ, Hezhou; WZ, Wuzhou; YL, Yulin; NN, Nanning; GG, Guigang; QZ, Qinzhou; BH, Beihai; BS, Baise; CZ, Chongzuo; HC, Hechi.

### 3.2 Molecular network of CRF55_01B in China

**Figure 2:**
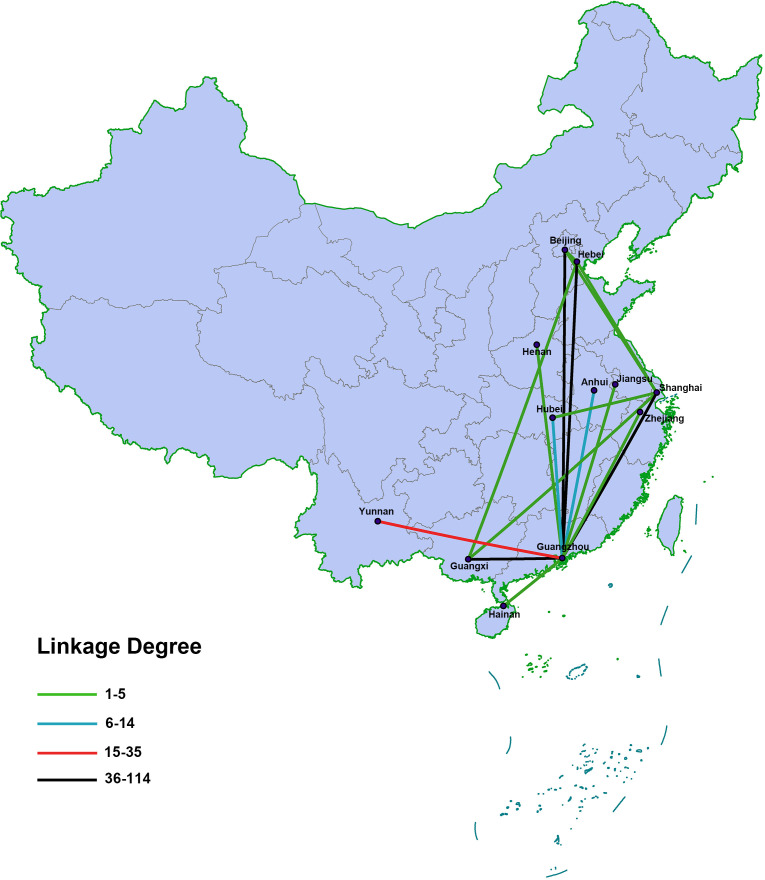
Linkage degree between different provinces in the molecular network. The color of the line segment corresponds to the linkage degree, defined as the number of degrees in a molecular network.

In total, 1418 CRF55_01B sequences were incorporated into the molecular network at a GD of 0.5%, forming 180 molecular clusters with a range of 2 to 715 nodes (**Supplementary Figure S1**). The largest molecular cluster consisted of 715 nodes and contained sequences from 11 provinces. As shown in **Figure 2**, the sequences from Guangdong Province played a pivotal role within the network by directly connecting with sequences from the other 11 provinces. Notably, the sequences from Guangxi, Beijing, Hebei Province, and Shanghai were highly correlated with those from Guangdong Province. The molecular network indicated that sequences from Guangdong Province, Hebei Province, and Shanghai were linked to Guangxi. Notably, sequences from Guangdong Province demonstrated the highest linkage degree with sequences from Guangxi.

### 3.3 Spatiotemporal transmission characteristics of CRF55_01B

**Figure 3:**
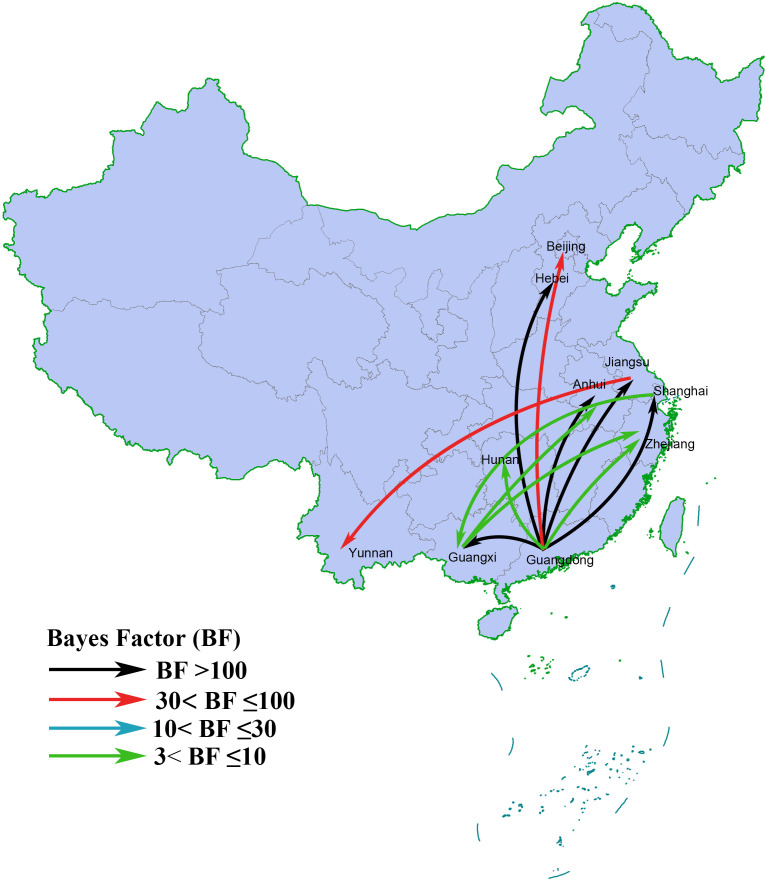
Transmission direction of CRF55_01B strain in China. The arrows represent the direction of transmission. The color of the line represents the size of the Bayes factor.

Phylogeographic analysis of the CRF55_01B sequences in China showed that this strain originated from Guangdong Province and that the estimated tMRCA was approximately 2003 (**Supplementary Figure S2**). The CRF55_01B strain was first introduced into Guangxi in 2007, and it then spread to other provinces after 2010. Guangdong Province served as the gateway for the CRF55_01B strain to spread to other regions of China (**Figure 3**). The regions in a transitional relationship with Guangxi were Guangdong Province, Shanghai, Anhui Province, and Zhejiang Province. Sequences from Guangdong Province and Guangxi exhibited a high degree of connectivity, with a BF of 74564 and a posterior probability of 1.0. Further details are shown in **Supplementary Table S3**.

### 3.4 Transmission dynamics across regions of Guangxi and/or Guangdong

**Figure 4:**
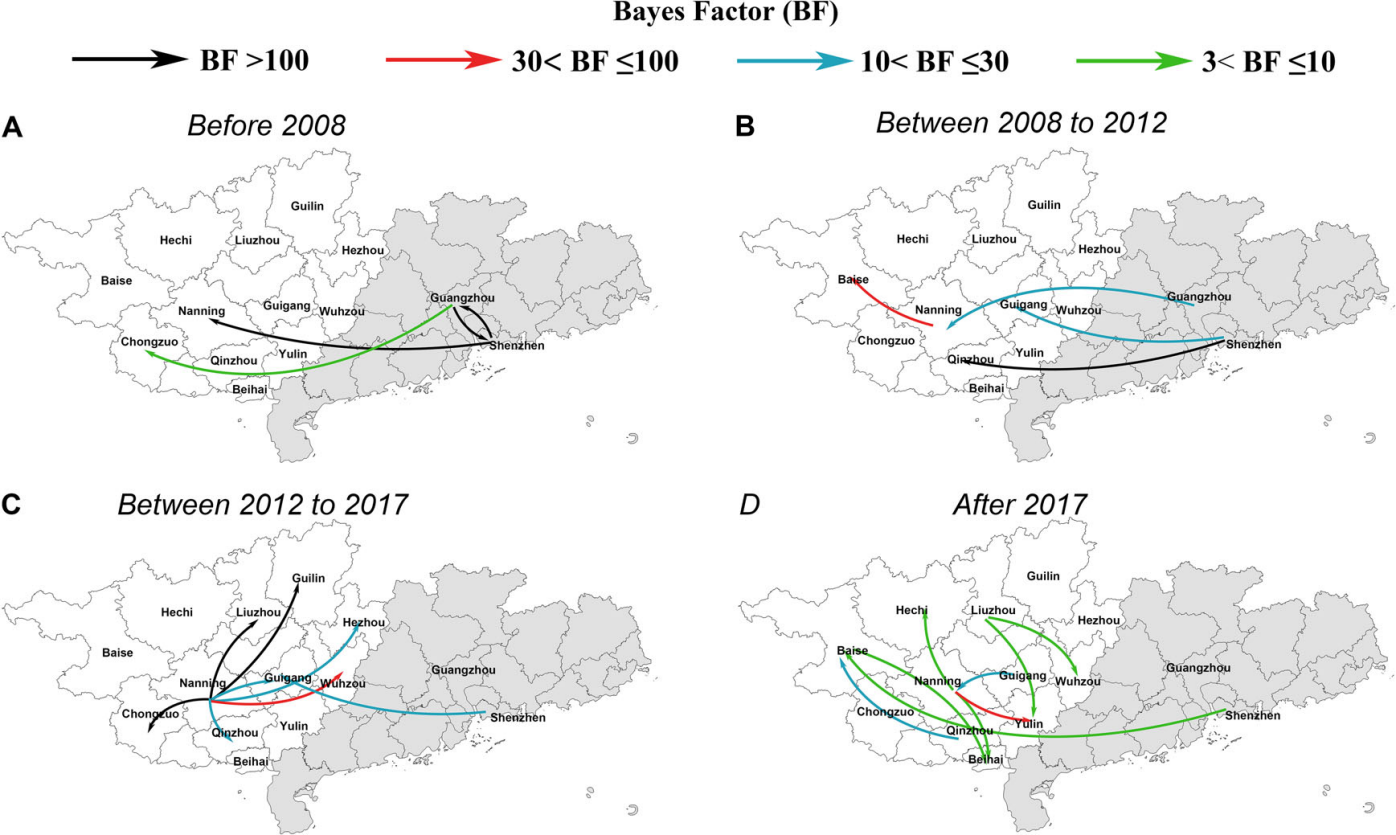
Migration events of CRF55_01B between regions in Guangdong and Guangxi through time: **(A)** Migration events before 2008. **(B)** Migration events between 2008 and 2012. **(C)** Migration events between 2012 and 2017. **(D)** and after 2017. The arrows represent the direction of transmission. The color of the line represents the size of the Bayes factor.

To elucidate the origin and transmission dynamics of CRF55_01B in Guangxi, we conducted distinct phylogenetic analyses utilizing sequences from Guangxi and Guangdong Province. The findings from the phylogeographical inference revealed a complex history of viral migration, highlighting numerous interconnections between Guangdong Province and Guangxi as well as intricate interactions across diverse regions within Guangxi (**Figure 4**).

These results indicate that the transmission of CRF55_01B was from Shenzhen City to Nanning City in 2007, substantiated by a BF of 221243 and a posterior probability of 1.0 (**Figure 4A**). Subsequently, the spread of CRF55_01B from Nanning City to other cities in Guangxi commenced in 2008 (**Figure 4B**), reaching its peak frequency of transmission between 2012 and 2017 (**Figure 4C**). After 2017, evidence of mutual viral transmission between distinct regions within Guangxi emerged. Notable migration events were supported by strong evidence, including transmissions from Nanning City to Chongzuo City, from Nanning City to Liuzhou City, and from Nanning City to Hechi City (**Figure 4D**). Nanning City emerged as a central hub facilitating the dissemination of CRF55_01B within Guangxi. Detailed data are provided in **Supplementary Table S4**.

### 3.5 Propagation characteristics of CRF55_01B among individuals with different sexual contact histories

**Figure 5:**
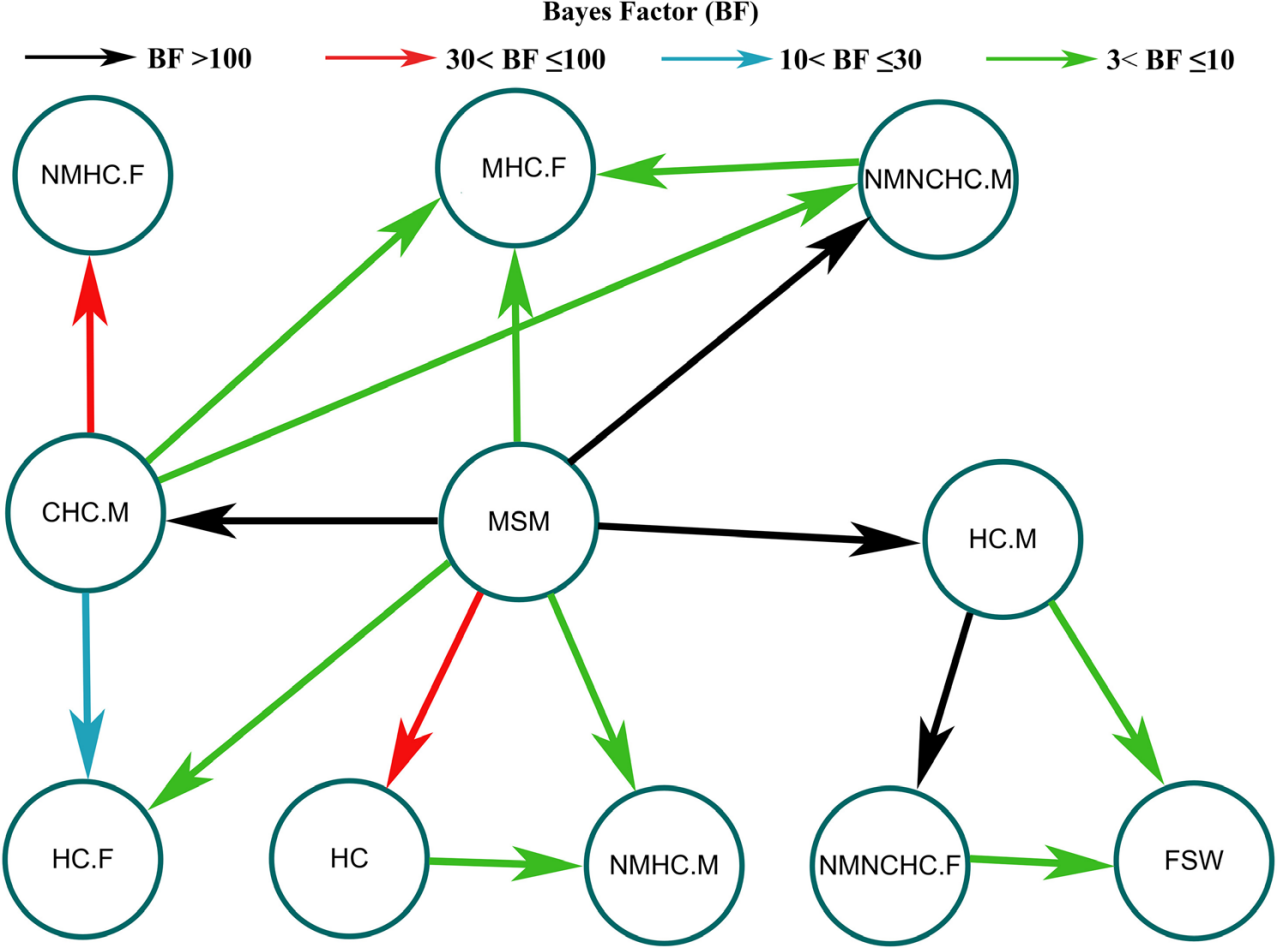
Transmission characteristics among groups with different sexual contact histories. The arrows represent the direction of transmission. The color of the line represents the size of the Bayes factor. NMHC.F, female with a history of non-marital heterosexual contact; CHC.M, men with a history of commercial heterosexual contact; HC.F, female with a history of heterosexual contact; MHC.F, female with a history of marital heterosexual contact; NMHC.M, men history of with non-marital heterosexual contact; HC.M, men with a history of heterosexual contact; NMNCHC.F, female with a history of non-marital non-commercial heterosexual contact.

A Bayesian model was employed to elucidate the correlation between the propagation of CRF55_01B and different sexual contact histories. As depicted in **Figure 5**, MSM emerged as the primary population driving the dissemination of CRF55_01B in Guangxi. The findings of this study indicate a high probability that CRF55_01B was transmitted from MSM to men with an NMNCHC history (BF=278.93, posterior probability=0.97), to men with a CHC history (BF=16707.25, posterior probability=1.0), and to men with an HC history (BF = 1276.60, posterior probability = 0.99).

Furthermore, our analysis revealed that women played a passive role in the transmission of CRF55_01B in Guangxi. Most infections in women could be traced back to men. Specifically, transmission to women with an NMNCHC history occurred through men with an HC history (BF = 1276.60, posterior probability = 0.99). Similarly, transmission to women with an NMHC history occurred through men with a CHC history (BF = 35.53, posterior probability = 0.79). Detailed data are provided in **Supplementary Table S5**.

## 4 Discussion

In this study, we elucidated for the first time the spatiotemporal transmission dynamics of CRF55_01B in different regions of Guangxi and the transmission patterns among populations with different sexual contact histories using a molecular network and phylogeographic and phylodynamic methods. Our findings demonstrated that CRF55_01B is extensively disseminated throughout Guangxi. The most probable source of transmission is Guangdong Province, with an estimated tMRCA of 2009. We determined that MSM and men with a history of CHC have played a key role in the dissemination of CRF55_01B in Guangxi.

We found that the CRF55_01B strain disseminated from MSM to other populations through diverse transmission routes, reflecting the intricate sexual contact relationship among individuals infected with CRF55_01B. Given that MSM and men with a CHC history have been identified as high-risk groups for second-generation transmission of HIV-1, it is imperative to strengthen the monitoring for MSM and men with a CHC history.

In addition, this study observed that CRF55_01B originated in 2003 from Guangdong Province and disseminated into Guangxi in 2007, which is consistent with previous findings (Zai et al., 2020; Gan et al., 2021). CRF55_01B was first identified in Guangdong, where it resulted in an outbreak and disseminated to other regions in China (Han et al., 2013; Zhao et al., 2014; Zai et al., 2020; Gan et al., 2021). Guangdong has the largest migrant population in China. Given that Guangxi shares a border with Guangdong Province and is the largest migrant population that has moved to Guangdong (Network, 2023), Guangdong is likely the primary source of the CRF55_01B strain prevalent in Guangxi. This study did not find evidence indicating that the CRF55_01B strain has spread from Guangxi to other provinces in China.

The Bayesian analysis suggested that CRF55_01B was first introduced into Nanning City from Shenzhen City in 2007 and subsequently spread from Nanning City to other cities within Guangxi. It is not uncommon for Chinese residents to travel to near metropolises for trade or work-related activities. Nanning City is a preferred destination for Guangxi residents engaged in trade or work because it serves as the economic and political center of Guangxi. Additionally, Nanning City is the region in Guangxi with the most severe HIV/AIDS epidemic (Lan et al., 2021). These factors have accelerated the dissemination of the CRF55_01B strain from Nanning City to other urban centers in Guangxi.

Previous studies have shown that MSM represent a key group affected by CRF55_01B infection (Zhao et al., 2014; Zai et al., 2020; Gan et al., 2021). However, our findings indicate that more than half of individuals infected with the CRF55_01B strain had a history of HC in Guangxi. Additionally, CHC and NCNMHC exhibited the highest prevalence within the HC group. There are two potential reasons for these findings. First, the primary mode of HIV-1 transmission among infected individuals in Guangxi is HC, particularly CHC and NCNMHC (Dong et al., 2021). MSM transmission accounts for only 7% of HIV-1 cases, which is significantly lower than the national average of 23% in China and 60% in Guangdong Province (Daily, 2016; He, 2021; Lan et al., 2021). Second, some men might conceal their same-sex behaviors because of a perceived higher level of discrimination, thus reporting a history of HC. Therefore, the proportion of individuals infected with CRF55_01B through HC in Guangxi might be overestimated.

In this study, we constructed a transmission pathway of the CRF55_01B strain among individuals with different sexual contact histories. The findings indicated that MSM played a predominant role in transmitting the CRF55_01B strain to other populations in Guangxi. Considering that the CRF55_01B strain originated from MSM and spread outward through this population (Gan et al., 2021), this finding is not unexpected. It is well acknowledged that MSM represent a high-risk group for the transmission of HIV-1. Previous studies have indicated that MSM in China exhibit lower rates of condom use, engage in multiple sexual partners, and might be identified as bisexual (Zhao et al., 2021). Because same-sex marriage is illegal in China, a significant number of MSM may engage in HET activity and marry women (i.e., MSMW). MSMW serve as a ‘bridge’ for the transmission of HIV-1 from MSM, a high-risk group, to women, a low-risk group (Tao et al., 2013; Oster et al., 2015). Furthermore, research indicates that compared with MSM only, MSMW are more likely to engage in unprotected and commercial sex (Tao et al., 2013; Wang et al., 2015). Therefore, it is essential to actively promote respect and social equality for MSM, with a particular focus on MSMW.

Our study also revealed that women represent the terminal end of the CRF55_01B transmission chain, whereas men engaged in commercial HET activities represent the main source of transmission of the CRF55_01B strain to women. This suggests a significant contribution of CHC to the dissemination of CRF55_01B in Guangxi. The primary mode of HIV-1 transmission among infected individuals in Guangxi is through CHC (Dong et al., 2021). Men with a history of CHC have a higher risk of HIV-1 infection and transmission (Kaufman, 2011). Our earlier research showed that men with CHC were responsible for the majority of local HIV-1 transmissions and were prone to transmitting the virus to other populations (Chen et al., 2021). Many patrons are reluctant to use male condoms because of difficulties with erection or discomfort, and this limits the promotion of condom use in sexual transactions. Prospective studies suggest that the provision of female condoms can reduce the incidence of unprotected sexual intercourse (Beksinska et al., 2019; Zhang et al., 2020). Therefore, promoting the use of female condoms may be an effective strategy for the prevention of HIV-1 transmission.

This study had several limitations. First, a relatively short sequence was used for the phylogenetic analysis. Shorter sequences cannot represent the dynamic evolution of the whole sequence, and this may have led to some bias of the results. Second, the non-Guangxi sequences were derived from the LANL HIV Database, which has been uploaded by other researchers. Therefore, these sequences may have been subject to publication bias, potentially leading to underestimation of the dissemination of CRF55_01B sequence in China. Third, the exclusive reliance on self-reported sexual contact histories may have led to recall bias and social desirability bias. Some individuals who reported having a history of HC were unable to further specify which type of HC they had (MHC, NMNCHC, or NMCHC). This might have diminished the reliability of the results regarding the transmission relationships among individuals with different exposure histories. Fourth, the accuracy of our results may have been affected by the quantity and quality of the sequence database. We downloaded all available *pol* sequences of the CRF55_01B strain, incorporated all CRF55_01B sequences sampled by our laboratory, and performed strict quality control prior to analysis.

## 5 Conclusion

This study provides the first estimation of the dissemination of the CRF55_01B strain from high-risk groups to the general population in Guangxi. MSM and men with CHC serve as a ‘bridge’ for the transmission of CRF55_01B, and studies of these populations provide valuable insights into prevention and control efforts. Furthermore, Nanning City was found to be the center of the dissemination and proliferation of the CRF55_01B strain to other cities in Guangxi. Considering the complex relationship between HIV-infected individuals with different sexual contact histories in Guangxi, it is imperative to adopt a multifaceted approach to mitigate the second-generation transmission of CRF55_01B, especially among MSM and men with a history of CHC.

## Data Availability

The datasets presented in this study can be found in online repositories. The names of the repository/repositories and accession number(s) can be found below: https://www.ncbi.nlm.nih.gov/genbank/, 2hzDLVrHHT.
