# Supplementary material for: Men with a history of commercial heterosexual contact play essential roles in the transmission of HIV-1 CRF55_01B from men who have sex with men to the general population in Guangxi, China
Source: Front Cell Infect Microbiol. 2024 Aug 23;14:1391215. doi: 10.3389/fcimb.2024.1391215 (PMC11377415; doi:10.3389/fcimb.2024.1391215)
Supplement: Supplementary file 2 [file DataSheet2.docx]

***Supplementary Material***

**Table S1.** Sample years and locations of sequences used in this study

| **Variables** | **Total, n (%)** |
| --- | --- |
| **Sample year** |  |
| 2007 | 3 (0.13) |
| 2008 | 24 (1.08) |
| 2009 | 54 (2.43) |
| 2010 | 46 (2.07) |
| 2011 | 87 (3.91) |
| 2012 | 186 (8.36) |
| 2013 | 215 (9.66) |
| 2014 | 269 (12.08) |
| 2015 | 281 (12.62) |
| 2016 | 186 (8.36) |
| 2017 | 308 (13.84) |
| 2018 | 286 (12.85) |
| 2019 | 168 (7.55) |
| 2020 | 32 (1.44) |
| 2021 | 40 (1.80) |
| 2022 | 41 (1.84) |
| **Location, province or city in China** |  |
| Anhui | 17 (0.76) |
| Beijing | 4 (0.18) |
| Chongqing | 1 (0.04) |
| Guangdong | 1894 (85.09) |
| Guangxi | 199 (8.94) |
| Harbin | 1 (0.04) |
| Hainan | 8 (0.36) |
| Hebei | 11 (0.49) |
| Henan | 7 (0.31) |
| Hubei | 15 (0.67) |
| Hunan | 2 (0.09) |
| Jiangsu | 21 (0.94) |
| Jiangxi | 1 (0.04) |
| Shanghai | 24 (1.08) |
| Shaanxi | 1 (0.04) |
| Yunnan | 14 (0.63) |
| Zhejiang | 6 (0.27) |
| **Transmission route** |  |
| HET | 195 (8.76) |
| IDU | 9 (0.40) |
| MSM | 767 (34.46) |
| Unknown | 1255 (56.38) |

**Abbreviations:** HET, heterosexual; IDU, injecting drug user; MSM, men who have sex with men.

**Table S2.** Sample years and locations of sequences obtained in Guangxi

| **Variables** | **Total, n (%)** |
| --- | --- |
| **Sample year** |  |
| 2009 | 1 (0.50) |
| 2012 | 2 (1.01) |
| 2013 | 4 (2.01) |
| 2014 | 1 (0.50) |
| 2016 | 2 (1.01) |
| 2017 | 19 (9.55) |
| 2018 | 24 (12.06) |
| 2019 | 46 (23.12) |
| 2020 | 30 (15.08) |
| 2021 | 29 (14.57) |
| 2022 | 41 (20.60) |
| **Sample region** |  |
| Baise | 7 (3.52) |
| Beihai | 1 (0.50) |
| Chongzuo | 29 (14.57) |
| Guigang | 25 (12.56) |
| Hechi | 1 (0.50) |
| Hezhou | 2 (1.01) |
| Liuzhou | 12 (6.03) |
| Nanning | 89 (44.72) |
| Qinzhou | 27 (13.57) |
| Wuzhou | 5 (2.51) |
| Yulin | 1 (0.50) |


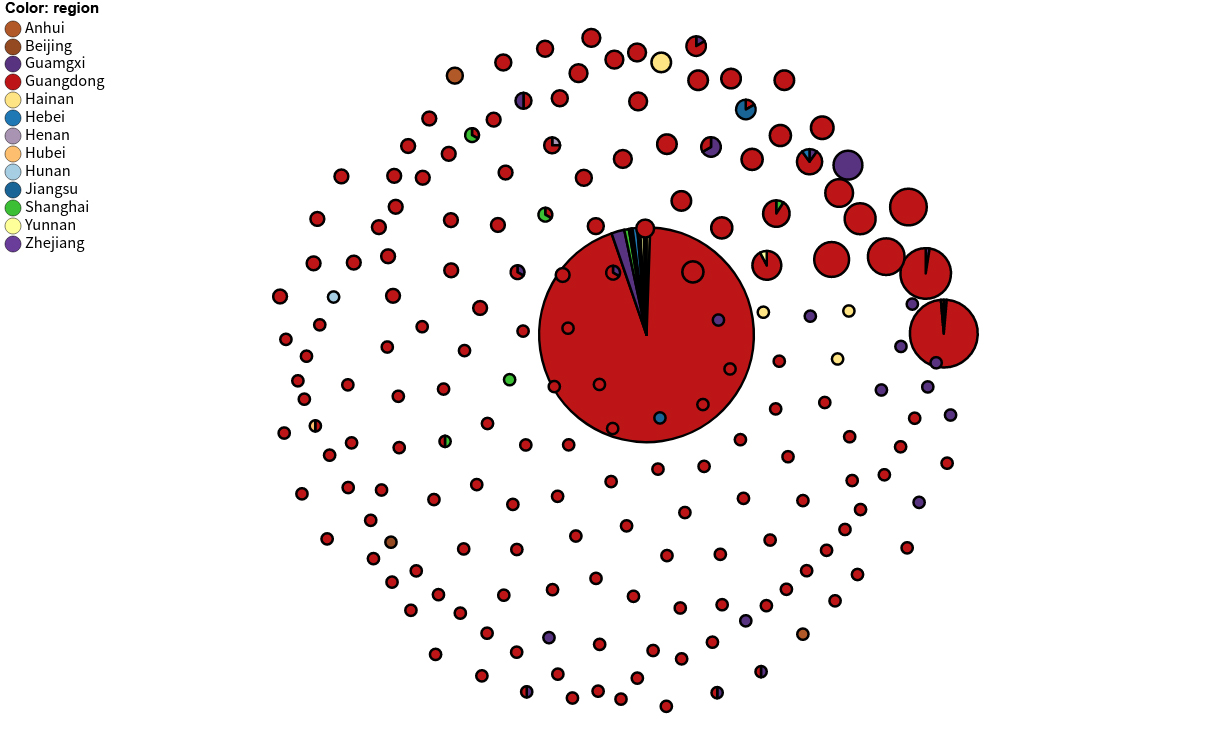


**Figure S1.** Molecular network diagram of CRF55_01B strain.

**Table S3.** Results of BSSVS in different regions of China

| From | To | Bayes factor* | Posterior probability |
| --- | --- | --- | --- |
| Guangdong | Guangxi | 74564.19075 | 1 |
| Guangdong | Shanghai | 74564.19075 | 1 |
| Guangdong | Jiangsu | 879.4826573 | 0.990667704 |
| Guangdong | Anhui | 802.2854776 | 0.989778913 |
| Guangdong | Hebei | 132.4178742 | 0.941117654 |
| Guangdong | Beijing | 77.03828862 | 0.902899678 |
| Jiangsu | Yunnan | 40.87295126 | 0.831463171 |
| Guangxi | Zhejiang | 9.114174299 | 0.523830685 |
| Guangdong | Zhejiang | 7.209059512 | 0.465281635 |
| Guangdong | Hunan | 6.334277461 | 0.433285191 |
| Shanghai | Guangxi | 3.803349871 | 0.314631708 |
| Guangxi | Anhui | 3.205455966 | 0.278969003 |

*The transmission relationships with a Bayes factor of >3 were selected.

BSSVS, Bayesian stochastic search variable selection





**Figure S2.** Maximum clade credibility tree of CRF55_01B strain in China. The maximum clade credibility tree was constructed using BEAST with the GTR substitution model, an uncorrelated relaxed clock model, and the Bayesian Skyline model through Bayesian inference.

**Table S4.** Results of BSSVS between Guangdong and Guangxi

| From | To | Bayes factor | Posterior probablity |
| --- | --- | --- | --- |
| Guangzhou | Shenzhen | 221243.7587 | 1 |
| Shenzhen | Nanning | 221243.7587 | 1 |
| Nanning | Liuzhou | 7889.710467 | 0.998444531 |
| Shenzhen | Qinzhou | 1878.786031 | 0.993500361 |
| Shenzhen | Guangzhou | 468.7000932 | 0.974445864 |
| Nanning | Guilin | 137.3070912 | 0.917837898 |
| Nanning | Chongzuo | 133.7522774 | 0.915838009 |
| Nanning | Baise | 82.74993867 | 0.870673851 |
| Nanning | Yulin | 70.39031312 | 0.851341592 |
| Nanning | Wuzhou | 57.17682765 | 0.823065385 |
| Shenzhen | Guigang | 27.2964507 | 0.689517249 |
| Guangzhou | Nanning | 22.60711068 | 0.647797345 |
| Nanning | Hezhou | 19.14156117 | 0.608966169 |
| Guigang | Nanning | 15.66622999 | 0.560357758 |
| Nanning | Qinzhou | 15.13938303 | 0.551913783 |
| Qinzhou | Baise | 12.88001682 | 0.511693795 |
| Nanning | Hechi | 8.04845301 | 0.395700239 |
| Shenzhen | Chongzuo | 7.032352563 | 0.363924226 |
| Shenzhen | Baise | 5.549032437 | 0.311038276 |
| Baise | Beihai | 5.538968574 | 0.310649408 |
| Nanning | Beihai | 5.416247101 | 0.305871896 |
| Liuzhou | Wuzhou | 4.99430898 | 0.288928393 |
| Nanning | Guigang | 4.428701989 | 0.264874174 |
| Liuzhou | Yulin | 3.627526753 | 0.227876229 |
| Guangzhou | Chongzuo | 3.082224404 | 0.200488862 |

*Transmission relationships with a Bayes factor of >3 were selected.

BSSVS, Bayesian stochastic search variable selection

**Table S5.** Results of BSSVS in the contact history

| From | To | Bayes factor | Posterior probability |
| --- | --- | --- | --- |
| MSM | CHC.M | 16707.24486 | 1 |
| MSM | HC.M | 16707.24486 | 1 |
| HC.M | NMNCHC.F | 1276.600101 | 0.992777778 |
| MSM | NMNCHC.M | 278.9291037 | 0.967777778 |
| MSM | HC | 60.36525379 | 0.866666667 |
| CHC.M | NMHC.F | 35.52947707 | 0.792777778 |
| CHC.M | HC.F | 21.21765798 | 0.695555556 |
| NMNCHC.M | MHC.F | 6.633544372 | 0.416666667 |
| CHC.M | MHC.F | 6.379890567 | 0.407222222 |
| CHC.M | NMNCHC.M | 4.855619789 | 0.343333333 |
| MSM | HC.F | 4.64348106 | 0.333333333 |
| HC | NMHC.M | 4.539780491 | 0.328333333 |
| NMNCHC.F | CHC.F | 3.875661358 | 0.294444444 |
| MSM | MHC.F | 3.310273612 | 0.262777778 |
| MSM | NMHC.M | 3.141686071 | 0.252777778 |
| HC.M | CHC.F | 3.086488521 | 0.249444444 |

*Transmission relationships with a Bayes factor of >3 were selected.

BSSVS, Bayesian stochastic search variable selection


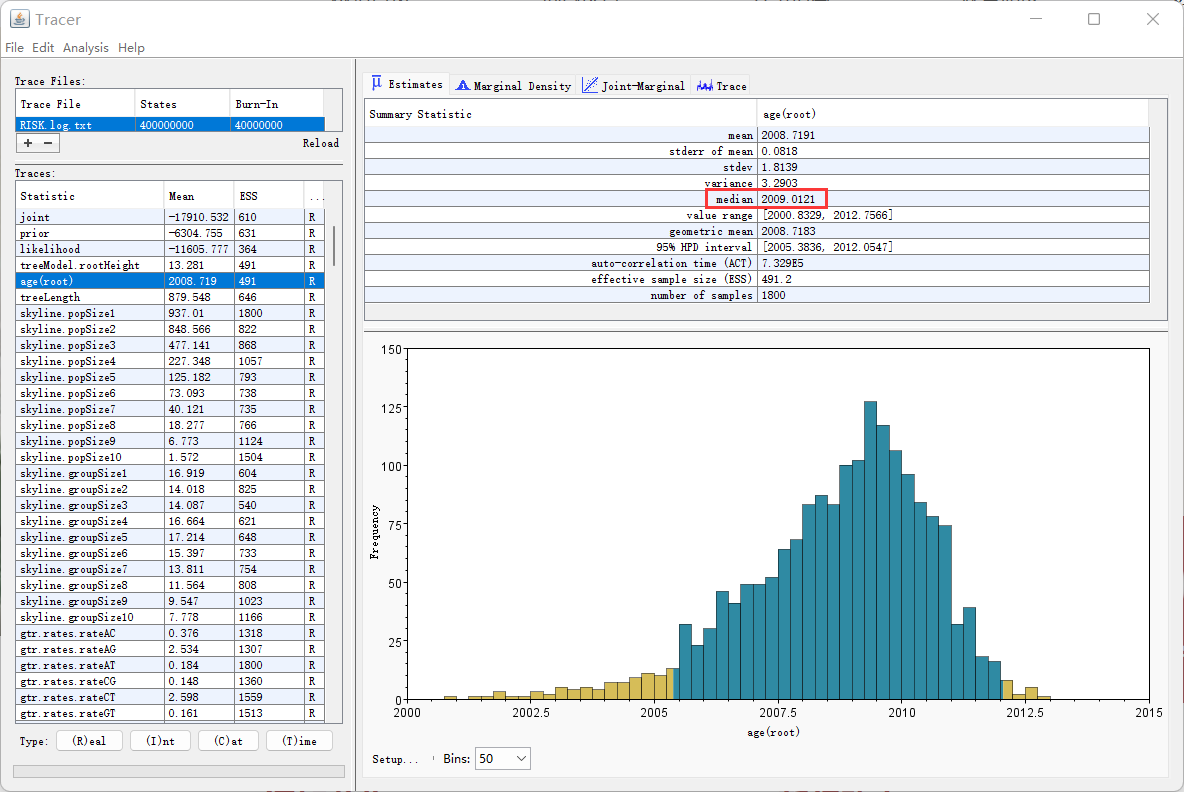


**Figure S3.** Analysis of the most recent common ancestor for Guangxi CRF55_01B.
